# Label-free quantitative proteomic analysis of the inhibition effect of *Lactobacillus rhamnosus* GG on *Escherichia coli* biofilm formation in co-culture

**DOI:** 10.1186/s12953-021-00172-0

**Published:** 2021-03-09

**Authors:** Huiyi Song, Ni Lou, Jianjun Liu, Hong Xiang, Dong Shang

**Affiliations:** 1 Clinical Laboratory of Integrative Medicine, First Affiliated Hospital of Dalian Medical University, 222 Zhongshan Road, Dalian, 116023 P. R. China; 2 Institute (College) of Integrative Medicine, Dalian Medical University, Dalian, China; 3 The Third Department of General Surgery, First Affiliated Hospital of Dalian Medical University, Dalian, Liaoning P. R. China

**Keywords:** Biofilm inhibition, Label-free quantitative proteomics, *Lactobacillus rhamnosus* GG microcapsules, *Escherichia coli*, Coculture

## Abstract

**Background:**

*Escherichia coli* (*E. coli*) is the principal pathogen that causes biofilm formation. Biofilms are associated with infectious diseases and antibiotic resistance. This study employed proteomic analysis to identify differentially expressed proteins after coculture of *E. coli* with *Lactobacillus rhamnosus* GG (LGG) microcapsules.

**Methods:**

To explore the relevant protein abundance changes after *E. coli* and *LGG* coculture, label-free quantitative proteomic analysis and qRT-PCR were applied to *E. coli* and LGG microcapsule groups before and after coculture, respectively.

**Results:**

The proteomic analysis characterised a total of 1655 proteins in *E. coli* K12MG1655 and 1431 proteins in the LGG. After coculture treatment, there were 262 differentially expressed proteins in *E. coli* and 291 in LGG. Gene ontology analysis showed that the differentially expressed proteins were mainly related to cellular metabolism, the stress response, transcription and the cell membrane. A protein interaction network and Kyoto Encyclopaedia of Genes and Genomes (KEGG) pathway analysis indicated that the differentiated proteins were mainly involved in the protein ubiquitination pathway and mitochondrial dysfunction.

**Conclusions:**

These findings indicated that LGG microcapsules may inhibit *E. coli* biofilm formation by disrupting metabolic processes, particularly in relation to energy metabolism and stimulus responses, both of which are critical for the growth of LGG. Together, these findings increase our understanding of the interactions between bacteria under coculture conditions.

**Supplementary Information:**

The online version contains supplementary material available at 10.1186/s12953-021-00172-0.

## Background

Biofilms are complex bacterial community structures that can attach to surfaces. They connect to a surface via extracellular polymeric substances (EPS), which form a matrix composed primarily of polysaccharides, proteins and DNA; this encapsulates the bacteria [1]. Biofilms not only cause economic losses but also present a public health hazard. This is because the bacteria present within biofilms are much more resistant to antibiotics, disinfectants [2] and host immune system effectors [3]. Therefore, it is critical to develop effective non-toxic—or less toxic—antifungal agents with novel modes of action.

A recent study suggested that probiotic supernatants have antibiofilm formation properties [4], which implies that probiotics may inhibit biofilm formation through cell-cell communication. However, there has been little progress in this field to date. In our previous study, bacteria immobilised in microcapsules showed superior biofilm inhibition capacity compared to probiotic sterile culture supernatant. Accordingly, a *Lactobacillus rhamnosus* GG (LGG) microcapsule–planktonic *Escherichia coli (E. coli)* coculture model was established to evaluate the biofilm inhibition effect [5]. However, the possible antibiofilm molecular mechanisms of LGG microcapsules have not yet been investigated.

Proteomic analysis for global protein identification is a powerful tool and has emerged as an important approach for extracting detailed information on cellular regulatory mechanisms at the protein level. Label-free quantitative proteomics provides a straightforward option for large-scale analysis of biological samples. In contrast to label-based methods, label-free quantitative proteomics has several advantages as it is cost-effective and does not require expensive labeling reagents. Also, label-free quantitative proteomics is not time-consuming compared to some label-based methods as these require tedious labeling steps [6]. For all these reasons, label-free quantitative proteomics has gained widespread acceptance in biomedical research, such as for the analysis of bodily fluids (blood, plasma, saliva, and urine), cell lines and tissues [7, 8].

In the present study, coculture experiments and proteomic analyses were performed to further advance understanding of such interactions and the potential underlying mechanisms. A label-free quantitative proteomic approach was used to identify proteins with significantly changed expression profiles during the *E. coli* and LGG microcapsule coculture process. Ultimately, these findings will contribute to an increased understanding of the possible molecular action of LGG microcapsules against *E. coli* biofilm formation and provide a powerful platform for future mechanistic studies of bacterial interactions.

## Materials and methods

### Bacterial strains and materials

LGG and *E. coli* K12MG165 were obtained from the American Type Culture Collection (ATCC 53103 and ATCC 47076). LGG was cultured in a modified MRS broth in which glucose was replaced by galactose under anaerobic conditions at 37 °C. *E. coli* strains were cultured at 37 °C in Luria-Bertani broth [9]. Cell suspensions were subsequently used as described below.

Sodium alginate was purchased from the Qingdao Crystal Salt Bioscience and Technology Corporation (Qingdao, Shandong, China). Chitosan was degraded from raw chitosan using the chemical method (Yuhuan Ocean Biomaterials Corporation, China). All other reagents and solvents were of analytical grade and were used without further purification.

### Preparation of LGG microcapsules

LGG alginate beads were prepared using the emulsification/internal gelation technique, as described previously [10]. Briefly, sodium alginate powder was dissolved in 0.9% (w/v) NaCl solution to obtain a final concentration of 1.5% (w/v). The cell pellet was obtained by centrifugation at 10,000 rpm for 5 min. The cells and micro-crystalline CaCO_3_ powder were finely dispersed in sterile sodium alginate solution. Then, the alginate-calcium salt-cell suspension and 200 mL of liquid paraffin containing 0.5% (v/v) Span 85 were stirred in a turbine reactor at 200 rpm for 30 min. After 30 min of emulsification, glacial acetic acid was added for gelification following which 500 mL of deionised water was added with stirring for 30 min at 200 rpm. The cell-entrapped calcium alginate beads with an initial cell number of about 1.0 × 10^6^ CFU/mL beads were then rinsed with 1% (v/v) Tween 80 solution and distilled water and were then stored in water at 4 °C.

### Preparation of LGG alginate–chitosan microcapsules

Chitosan solution was dissolved in 0.1 M acetate buffer. The cell-entrapped calcium alginate beads were immersed in 0.5% (w/v) chitosan solution by gently shaking at a bead/solution ratio of 1:5 (v/v). After rinsing and liquefication for 6 min using 0.055 M sodium citrate, the cell-entrapped alginate-chitosan microcapsules were formed.

### Characterisation of microcapsules

Microcapsule size was examined with a Counter Coulter LS130 particle size analyser, which has a size range of 0.1 to 1000 μm. Optical images were observed under a Nikon Eclipse TE2000 Inverted Research Microscope (Nikon Corp., Japan).

### Biofilm thickness detection by confocal microscopy

Biofilm thickness was detected according to a previously published method, with minor modification [11]. After incubation, the microscope slides with biofilm from each group were gently rinsed with deionised water to remove unattached cells and then stained with SYTO9/propidium iodide according to the instructions of the L13152 LIVE/DEAD BacLight bacterial viability kit (Invitrogen Molecular Probes, USA). After staining for 30 min, sterile PBS was used to remove the planktonic dyes and bacteria, and the stained coverslips were visualised under a confocal laser scanning microscope (Leica SP8, Germany) at an excitation wavelength of 488 nm and 200× magnification. Three-dimensional biofilm images were obtained using CLSM software. Image stacks of three random spots were collected from three sets of biofilm samples and saved in “tif” format.

### Preparation of cell samples and experimental grouping

Briefly, LGG microcapsules were cocultured with planktonic *E. coli* for 48 h for biofilm inhibition, as reported previously [5]. At the end of coculture, the biofilm was meticulously scraped off the well wall using a sterile scalpel. The *E. coli* cells in the biofilm were collected by sonication using high-intensity focused ultrasound (UTR2000, Hielscher); these were denoted as group A. Group A was lysed in medium containing 4% w/v sodium dodecyl sulphate (SDS), 0.1 mM dithiothreitol (DTT), and 100 mM Tris-HCl at pH 8.2. At the same time, the LGG microcapsules were collected after coculture and the entrapped LGG cells were released from the microcapsules according to a previously described method [12]; these were denoted as group B. *E. coli* pure culture (denoted as group C) and 48 h LGG microcapsules pure culture (denoted as group D) were used as the negative controls.

### Protein extraction and digestion

To the samples was added 1 mL of lysis buffer containing 8 M urea and 10% protease inhibitor and the resulting mixture carefully transferred 1.5 mL microfuge tubes. The samples were sonicated using a probe sonicator, then centrifuged at 14,000×g for 30 min, following which the supernatant was collected. The protein concentration was determined using the Bradford method and the remainder of the sample was frozen to − 80 °C.

From each sample, 50 μg of total protein was used for further analysis. The samples were diluted with ammonium bicarbonate (ABC) buffer, which allowed for a steady pH value of approximately 7.0 for eight times, and then reduced with 200 mM of DTT solution and incubated at 37 °C for 1 h. The samples were digested overnight with trypsin (trypsin:protein = 1:25) at 37 °C. After concentration using a Speedvac (Thermo Savant SPD121P, Thermo Scientific, Wohlen, Switzerland), each sample was reconstituted in 3% acetonitrile (ACN) and 0.1% formic acid (FA).

### Protein fractionation by reverse-phase liquid chromatography

The next day, 50 μL of 0.1% formic acid (FA) was added to the incubated mixture to terminate the digestion. A C18 column was washed with 100% ACN followed by 0.1% FA and centrifuged at 1200 rpm for 3 min. The Eppendorf (EP) tube was replaced and the sample (≤ 30 μg) was added and then centrifuged at 1200 rpm for 3 min. The samples were washed once with 100 μL of pH 10 water. After this process, the sample was eluted with 10 gradients of ACN in pH 10 water at the following concentrations: 6% (i.e. 60 μL ACN, 940 μL pH 10 water), 9, 12, 15, 18, 21, 25, 30, 35 and 50%. Next, the eluent from the 6, 12, 15, 25 and 35% gradients were combined in one tube while the 9, 18, 21, 30 and 50% gradients were combined in another tube, thus dividing the sample into two fractions. The sample fractions were then lyophilised and stored at − 80 °C until loading.

### Peptide identification by liquid chromatography with tandem mass spectrometry (LC-MS/MS)

Samples were injected onto an in-house pulled and packed tip column (length 8 cm) carrying Magic C18 AQ beads (3 μm bead size, 200 Å pore size; Bishoff Chromatography, Leonberg, Germany), 75 μm ID, 375 OD capillary, coupled to an Eksigent nanoLC-1D device (ABSciex, Zug, Switzerland). They were then separated using a binary solvent system with a flow rate of 200 nL/min, eluted using a gradient from 2 to 30% B over 60 min (A:1% ACN, 0.1% FA, B: 100% ACN, 0.1% FA) and acquired using an LTQ Orbitrap (ThermoScientific, Wohlen, Switzerland) equipped with a nanospray ion source running a standard collision-induced data-dependent (CID-DDA) method of one survey (MS) scan followed by ten dependent scans (MS/MS) looped throughout the run. The survey scan was acquired from 300 to 2000 m/z units in profile mode with a resolution of 30,000 in the Orbitrap. The dependent scans were acquired in centroid mode in the ion trap with a collision energy of 35, activation energy of 0.25 and 30 ms activation time, excluding singly charged ions for fragmentation. Dynamic exclusion was applied with a list size of 500 that was repeated every 30 s and with a duration of 90 s.

### Protein identification

The Uniprot_*Escherichia coli* (2019.4.20 download) database and the Uniprot_*Lactobacillus rhamnosus* (2019.7.25 download) database were used. The MS/MS data were processed using Maxquant 1.5.2.8 software and the identification parameters set with a precursor ion mass tolerance of ±15 ppm, fragment ion mass tolerance of ±0.5 Da, maximum of two missed cleavages, static modification with carboxyamidomethylation (57.021 Da) of the Cys residues, and dynamic modification with oxidation modification (+ 15.995 Da) of the Met residues. According to the primary data analyses, protein and peptide FDR were set to 0.01 (1%), the decoy database was set to revert and the minimum peptide number was 1 for the identified proteins. Data with *p* ≤ 0.05 and a difference ratio of ≥1.2 were selected for further analysis. The statistical analyses of the LC-MS/MS data were performed using Perseus (v1.4.1.3).

### Bioinformatic analysis of identified proteins and prediction of promoters in intergenic regions

A functional category gene enrichment test was performed using Blast 2GO to determine whether there was enrichment in any functional subcategories. The number of differentially expressed proteins was imported into IPA (Ingenuity Pathway Analysis) software and used to perform protein biological pathway analysis based on the Gene Ontology (GO) and UniProt databases.

### Quantitative real-time PCR (qRT-PCR) analysis

LGG microcapsules were cocultured with planktonic *E. coli* for 48 h. The cells were then pelleted by centrifugation at 5000 rpm for 5 min at 4 °C and then resuspended in 1 mL TRIzol (Invitrogen, Carlsbad, CA) for total RNA isolation, according to the manufacturer’s protocol. Residual genomic DNA was then removed by treating isolated RNA with a Turbo DNAfree kit (Ambion, Austin, TX). Then, cDNA was synthesised using the PrimeScript RT Master Mix (Takara), according to the manufacturer’s instructions. qRT-PCR amplifications were performed with at least three biological replicates using 2 × SYBR Premix Ex TaqTM II (DRR081A, Takara) with Stratagene MX3000P (Agilent Technologies, CA, USA). The housekeeping gene 16S rRNA was used as a control for normalisation. The qRT-PCR primers are provided in Table S1. The data were normalised against 16S rRNA and the *p*-values from Student’s t-tests are reported as follows: * ≤ 0.05, ** ≤ 0.01, and *** ≤ 0.001.

### Detection of antibiotic resistance

The antibiotic resistance of the *E. coli* coculture with LGG microcapsules was determined using the inhibition zone method [13]. The *E. coli* culture before and after coculture was used as the test strain and inoculated into LB solid medium at 1% of the total amount. After solidification, 1 cm diameter filter paper with 500 μg/mL, 50 μg/mL or 5 μg/mL ampicillin (Amp) was impregnated on the inoculated agar plates and incubated for 24 h at 37 °C. The blank medium was the negative control. After incubation, the diameter of the inhibition zone (accurate to 0.1 mm) was measured.

## Results

### The inhibition effect of LGG microcapsules on *E. coli* biofilm formation

**Fig. 1:**
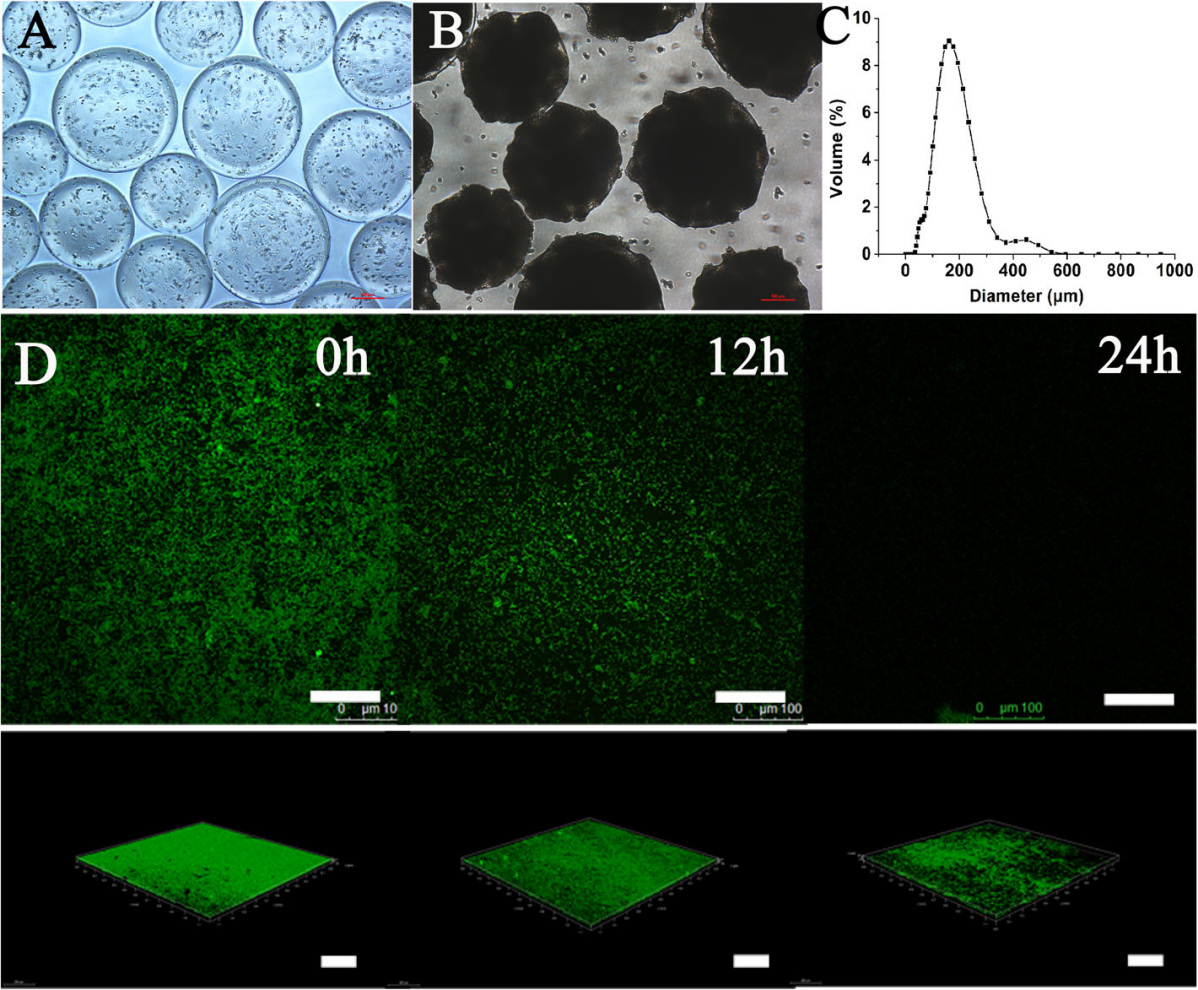
Optical images of LGG microcapsules produced by the emulsification/internal gelation technique (bar = 100 μm) at (**a**) 0 h culture and (**b**) 24 culture. **c** Size distribution of LGG microcapsules produced by the emulsification/internal gelation technique. **d** Confocal laser scanning microscopy images of *E. coli* biofilm formation after coculture with LGG microcapsules for 24 h

The LGG microcapsules appeared spherical, were well dispersed in the solution and were relatively uniform in size. Because of the high cell count of the microcapsules at the end of culturing, which resulted in the appearance of dark microcapsule density, the cells occupied almost all of the inner space (Fig. 1 a and b). The diameter of the microcapsules entrapped with LGG was about 200 μm (Fig. 1c). The observed reduction in the intensity of fluorescence provided evidence of biofilm inhibition, with the confocal software analysis showing that the *E. coli* biofilm thickness was decreased from 25.2 ± 1.3 μm to 12.3 ± 0.9 μm after coculture (Fig. 1d).

### Global proteomic analysis of *E. coli* and LGG before and after coculture treatment

**Fig. 2:**
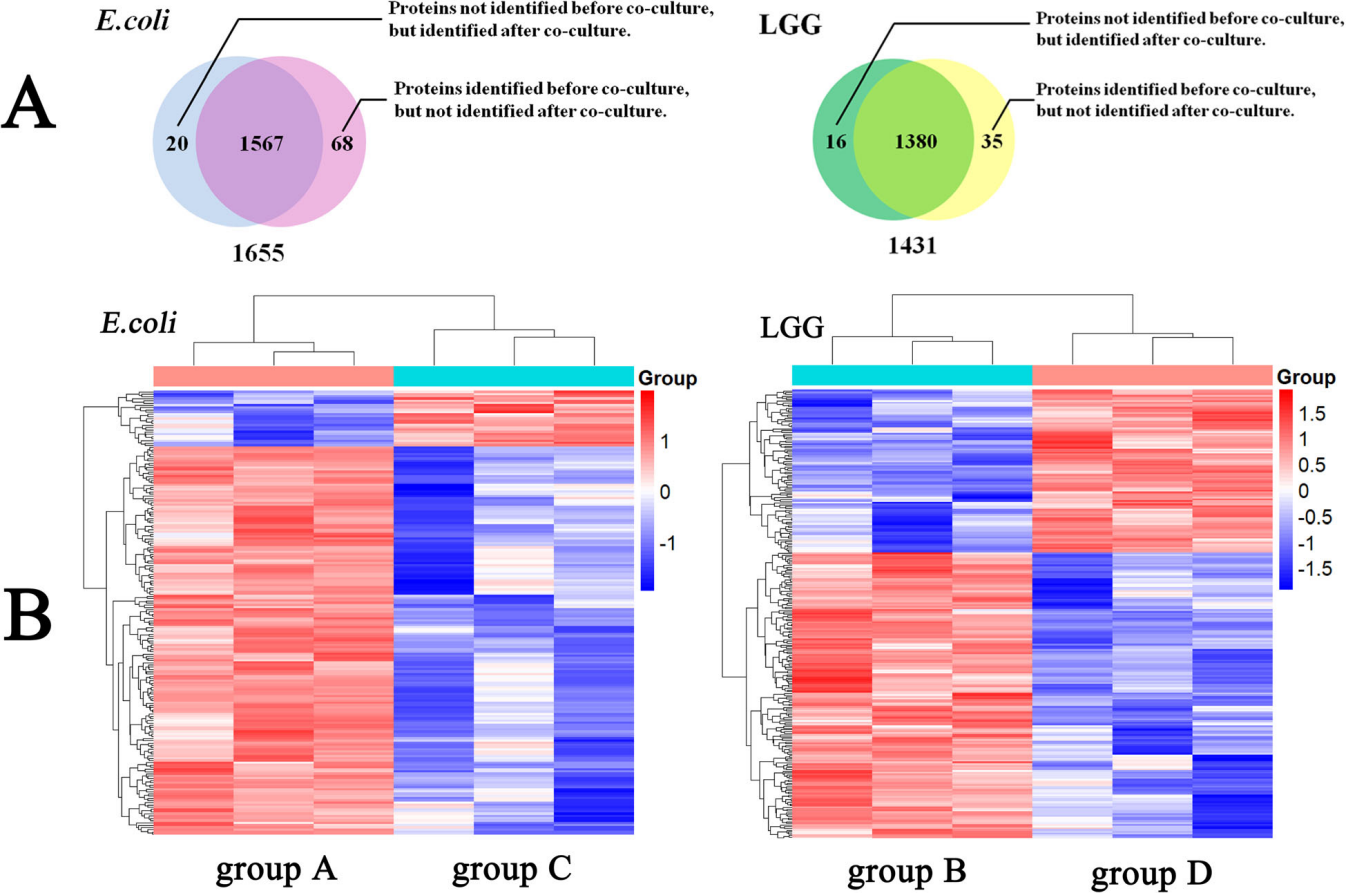
**a** Venn diagram summarising the common and differentially expressed proteins before and after coculture. **b** Hierarchical cluster of proteins differentially expressed in (a) *E. coli* before coculture (group C) and after coculture (group A), and (b) LGG before coculture (group D) and after coculture (group D). Red represents high expression and blue represents low expression. Two main clusters of proteins can be observed, one upregulated (right) and the other downregulated (left)

*E. coli* in the biofilm functional genome was evaluated at the proteome level in response to LGG microcapsules, before and after coculture. The experiments were performed in biological triplicates. Proteins from total bacterial lysates were extracted and digested in solution and the resulting peptides were analysed using LC-MS/MS [14]. In total, 76,382 matched spectra resulted in 12,236 matched peptides assembled into 1655 proteins in groups A and group C, and 58,028 matched spectra led to 10,321 matched peptides assembled into 1431 proteins in groups B and D (Additional files 1 and 2). Differential expression was considered as proteins that were significantly different with an ANOVA *p*-value < 0.05 and with at least a 1.2-fold change (cut-off value), as shown in the Venn diagram in Fig. 2a. For *E. coli*, there were 20 proteins not identified in group C but identified in group A, while 68 proteins identified in group C were not identified in group A. For LGG, there were 35 proteins not identified in group D but identified in group B, while there were 16 proteins identified in group D that were not identified in group B. Detailed information on these differentially expressed proteins is given in Additional files 3 and 4. Hierarchical cluster analysis was performed for the coculture and control groups. As shown in Fig. 2b, the common differentially expressed proteins in the coculture groups did not cluster with the ones in the control groups.

The next focus was on the common differentially expressed proteins identified both before and after coculture. As shown in Table 1 and Fig. S1, for *E. coli* a total of 262 differentially expressed proteins were identified in groups A and C, among which 31 proteins were upregulated and 231 proteins were downregulated. For LGG, 291 proteins were identified in groups B and D, among which 104 proteins were upregulated and 187 proteins were downregulated. The top 10 most differentially expressed proteins in both strains are shown in Tables 2 and 3.

**Table 1:** Number of common differentially expressed proteins that were modified 2-fold (up- or downregulation) in different experimental groups

Strains	Comparisons (Before coculture/After coculture)	
	Upregulated proteins	Downregulated proteins
*E.coli* (groupA+groupC)	31	231
LGG (groupB+groupD)	104	187

**Table 2:** Most highly common differentially upregulated and downregulated proteins in *E.coli*

**Upregulated protein**	**Protein ID**	***p*** **value**	**Log2FC**	**Protein description**
**bioD2**	P0A6E9	2.10E-05	1.71	ATP-denpendent dethiobiotin synthetase BioD2
**panD**	P0A790	5.20E-05	3.63	Aspartate 1-decarboxylase
**rpsP**	P0A7T3	2.83E-04	1.86	30S ribosomal protein S16
**hybC**	P0ACE0	3.31E-04	2.01	Hydrogenase-2 large chain
**rhIB**	P0A8J8	3.77E-04	1.06	ATP-dependent RNA helicase RhlB
**mlaC**	P0ADV7	4.10E-04	3.60	Intermembrane phospholipid transport system binding protein MlaC
**fimA**	P04128	5.11E-04	1.81	Type-1 fimbrial protein, A chain
**hdeA**	P0AES9	7.83E-04	1.17	Acid stress chaperone HdeA
**cysQ**	P22255	1.89E-03	1.41	3′(2′),5′-bisphosphate nucleotidase CysQ
**ygiW**	P0ADU5	2.06E-0	2.17	Protein YgiW
**Downregulated protein**	**Protein ID**	***P*** **value**	**Log2FC**	**Protein description**
**bamE**	P0A937	9.90E-04	−0.90	Outer membrane protein assembly factor BamE
**yejL**	P0AD24	1.29E-03	−3.09	UPF0352 protein YejL
**rpsN**	P0AG59	2.60E-03	−2.17	30S ribosomal protein S14
**gpmA**	P62707	4.76E-03	−0.52	2,3-bisphosphoglycerate-dependent phosphoglycerate mutase
**dnaK**	P0A6Y8	9.35E-03	−0.27	Chaperone protein DnaK
**sohB**	P0AG14	1.43E-02	−0.45	Probable protease SohB
**ychF**	P0ABU2	1.63E-02	−0.35	Ribosome-binding ATPase YchF
**guaB**	P0ADG7	2.64E-02	−0.97	Inosine-5′-monophosphate dehydrogenase
**ftsX**	P0AC30	3.38E-02	−2.46	Cell division protein FtsX
**ompA**	P0A910	4.93E-02	−1.08	Outer membrane protein A

**Table 3:** Most highly common differentially upregulated and downregulated proteins in LGG

**Upregulated protein**	**Protein ID**	***p*** **value**	**Log2FC**	**Protein description**
**CCE29_04955**	A0A1Y0DVK9	2.40E-05	1.76	Pilus assembly protein
**LRHMDP2_922**	K8QM21	4.30E-05	1.15	NADPH:quinine reductase related Zn-dependent oxidoreductase
**CCE29_07950**	A0A1Y0DXE7	9.90E-05	2.01	Iron-sulfur cluster biosynthesis family protein
**AAULR_10650**	F3N0G5	1.11E-04	3.68	Membrane protein
**CCE29_03735**	A0A2A5L8F8	1.23E-04	4.07	ABC transporter substrate-binding protein
**CCE29_04965**	A0A1Y0DVP1	2.67E-04	2.78	Pilus assemble protein
**N507_1524**	A0A249N5Y8	6.08E-04	0.80	Uncharacterized protein
**LRHMDP2_518**	K8QF03	7.93E-04	1.54	Uncharacterized protein
**purD**	A0A1Y0DZS2	1.60E-03	1.79	Phosphoribosylamine-glycine ligase
**purM**	A0A1Y0DZP0	1.63E-03	1.79	Phosphoribosylfomyglycinamidine cyclo-ligase
**Downregulated protein**	**Protein ID**	***p*** **value**	**Log2FC**	**Protein description**
**rpoC**	K8QEG2	8.00E-06	−0.55	DNA-directed RNA polymerase subunit beta′
**secA**	A0A249DE52	9.30E-05	−0.87	Protein translocase subunit SecA
**murB**	A0A3S4R547	2.81E-04	−2.82	UDP-N-acetylenolpyruvoylglucosa mine reductase
**murF**	A0A2A5L3G4	3.21E-04	−0.82	UDP-N-acetylmuramoyl-tripeptide-D-alanyl-D-alanine ligase
**rplV**	K8Q8J1	3.43E-04	−0.96	50S ribosomal protein L22
**ddl**	A0A1Y0DWH7	3.73E-04	−1.54	D-alanine-D-alanine ligase
**LRHMDP2_1796**	K8Q7G6	4.26E-04	−2.21	6-phospho-beta-glucosidase
**N507_1229**	A0A249N444	4.62E-04	−2.42	50S ribosomal protein L16
**ackA**	K8Q7E5	8.56E-04	−0.64	Acetate kinase
**lpdA**	A0A1Y0DTI9	1.75E-03	−1.08	Outer membrane protein A

### Functional categorisation of common differentially expressed proteins in *E. coli* and LGG

The possible functions of the common differentially expressed proteins were investigated. The functions of the regulated bacterial proteins were enriched according to GO terms, with the redundant GO terms summarised and unified.

#### Functional categorisation of common differentially expressed *E. coli* proteins

**Fig. 3:**
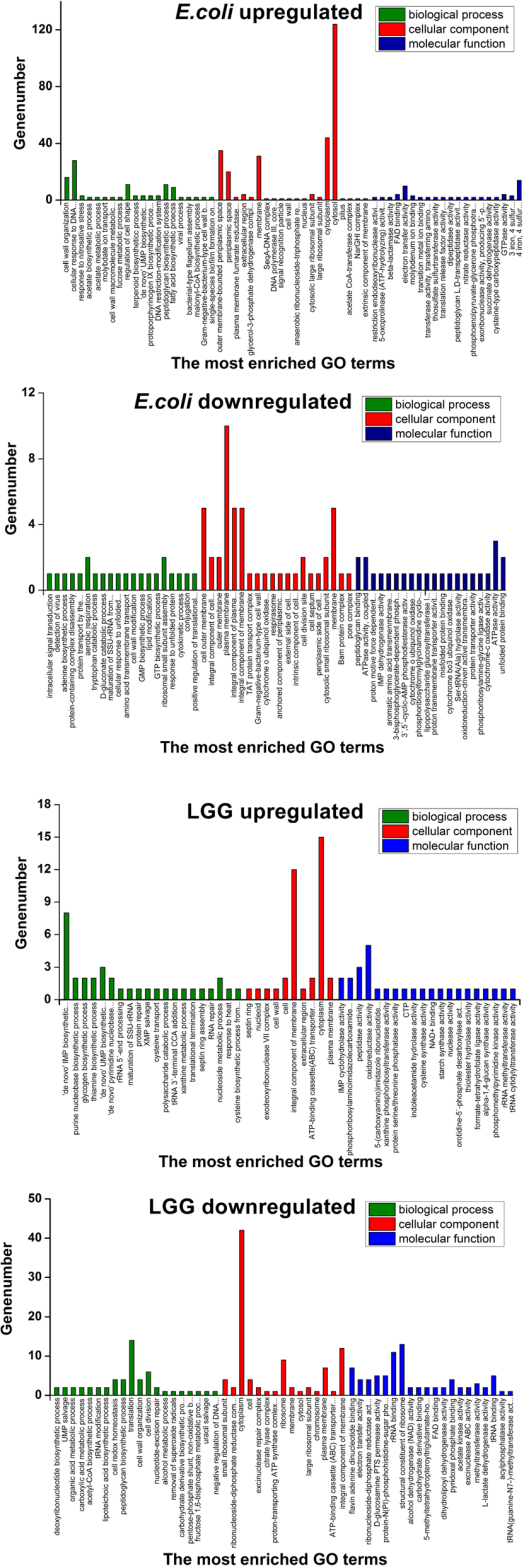
Annotation of overall regulated bacterial protein functions by enrichment of Gene Ontology (GO) terms. Based on the classifications of the GO annotation, the overall bacterial functions were categorised into biological processes, molecular functions, and cellular components, and are displayed in histogram format. The number of GO terms for each of the three categories is shown and the proportion of each specific subcategory is also provided. Subcategories with GO terms less than 1% are classified as “other”

A total of 358, 356 and 50 GO terms relating to molecular function, biological processes and cellular components, respectively, were generated based upon the upregulated proteins in *E. coli* after coculture; a total of 56, 57 and 26 GO terms, respectively, were generated based upon the downregulated proteins. Among the GO terms for biological processes, “cellular response to DNA damage stimulus” (22.75%) was the most common function in upregulated proteins in the *E. coli* coculture group. Among the GO terms for cellular components, the most common upregulated proteins belonged to “cytosol” (33.86%), while the most common downregulated proteins belonged to “plasma membrane” (12.58%). For molecular function, 13.51% of up- and 11.69% of downregulated proteins were related to “4 iron, 4 sulphur cluster binding” and “ATPase activity”, respectively (Fig. 3).

#### Functional categorisation of common differentially expressed LGG proteins

A total of 211, 138 and 20 GO terms for molecular function, biological processes and cellular components, respectively, were generated: 85, 50 and 10 GO terms, respectively, were related to upregulated proteins in LGG after coculture, while 150, 102 and 15 GO terms, respectively, were related to downregulated proteins. Among the GO terms for biological processes, “carbohydrate metabolic process” (22.75%) was the most common function in upregulated proteins in the coculture group. Among the GO terms for cellular components, the most common upregulated proteins belonged to “cytoplasm” (33.86%), while the most common downregulated proteins belonged to “ATP binding” (12.58%). For molecular function, 17.92% of up- and 16.55% of downregulated proteins were related to “oxidoreductase activity” and “structural constituent of ribosome”, respectively (Fig. 3).

### Pathway analysis

**Fig. 4:**
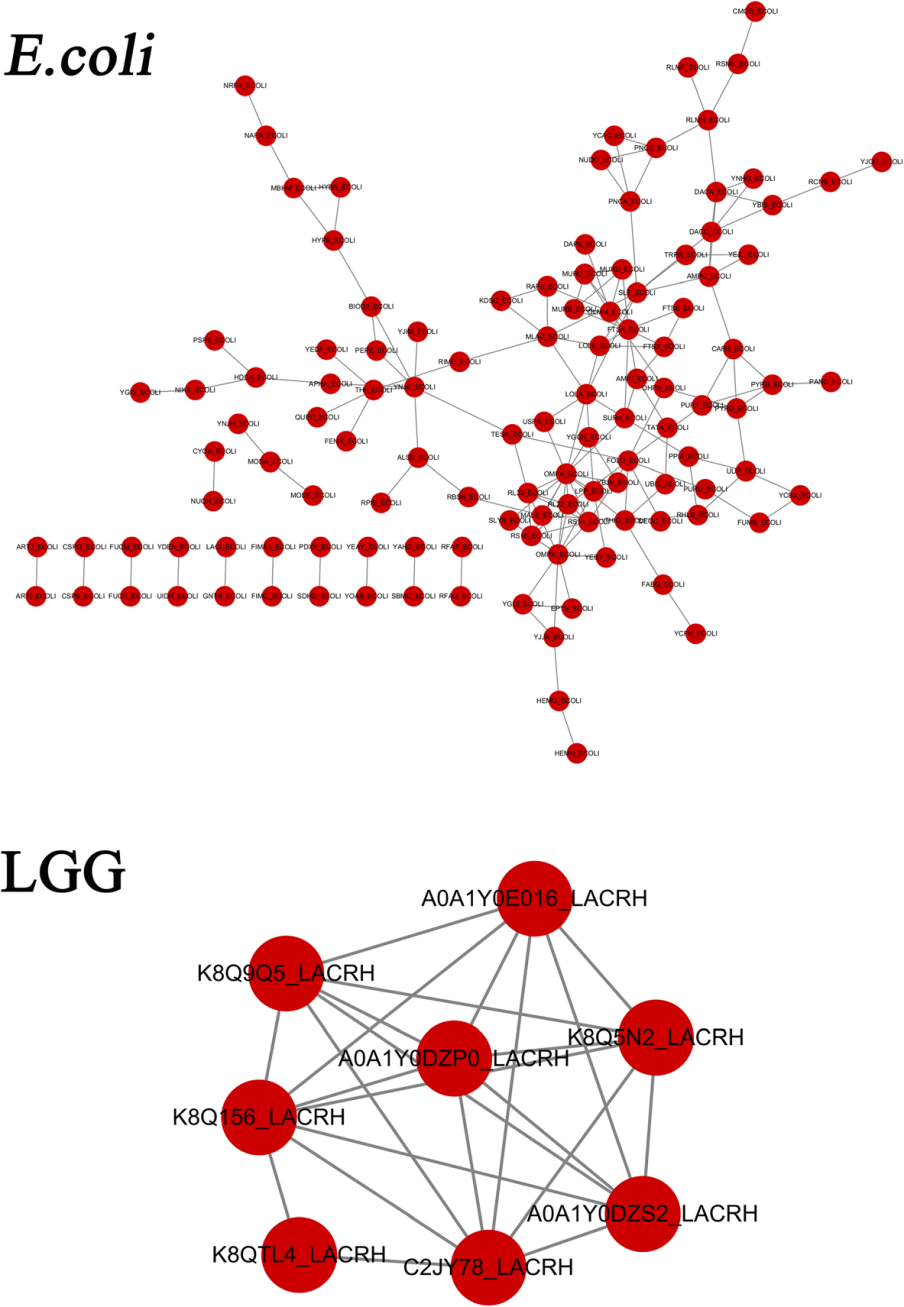
Protein-protein interaction networks in *E. coli* and LGG, respectively

As shown in Table 4, in *E. coli* the common differentially expressed upregulated proteins were enriched in the fatty acid biosynthesis, biotin metabolism and nitrogen metabolism pathways and the common differentially expressed downregulated proteins were enriched in the purine metabolism, oxidative phosphorylation and protein export signaling pathways. However, in LGG strains, as shown in Table 5, there were no upregulated pathways enriched in the KEGG analysis, and the common downregulated signaling pathways were galactose metabolism, amino sugar and nucleotide sugar metabolism, and metabolic pathways. By exploring the possible global protein-protein interactions (PPIs; Fig. 4), and in combination with Tables 2 and 3, several genes in *E. coli* and LGG were identified for subsequent analysis.

**Table 4:** Common pathway in *E.coli* groups

Pathway	***p*** value	Enrichment
**Up-enrichment**		
Fatty acid biosynthesis	0.0257	1.5887
Biotin metabolism	0.0502	1.2987
Nitrogen metabolism	0.0535	1.2716
Taurine and hypotaurine metabolism	0.0636	1.1959
Fatty acid metabolism	0.0664	1.1775
Peptidoglycan biosynthesis	0.0664	1.1775
Glutathione metabolism	0.1295	0.8875
Pyruvate metabolism	0.1478	0.8301
Terpenoid backbone biosynthesis	0.1489	0.8269
Propanoate metabolism	0.1551	0.8093
**Down-enrichment**		
Purine metabolism	0.0398	1.3991
Oxidative phosphory	0.0827	1.0823
Bacterial secretion	0.1857	0.7310
Protein export	0.1857	0.7310
RNA degradation	0.1997	0.6996
Ribosome	0.2042	0.6898
Phosphotransferase	0.2661	0.5749
Galactose metabolism	0.2661	0.5749
Lipopolysacharide biosynthesis	0.2912	0.5357
Methane metabolism	0.3390	0.4697

**Table 5:** Common pathway in LGG groups

Pathway	***p*** value	Enrichment
**Down-enrichment**		
Galactose metabolism	1	0
Amino sugar and nucleotide sugar metabolism	1	0
Metabolic pathways	1	0

### Confirmation of the target proteins of *E. coli* and LGG at the mRNA level in the coculture model

**Fig. 5:**
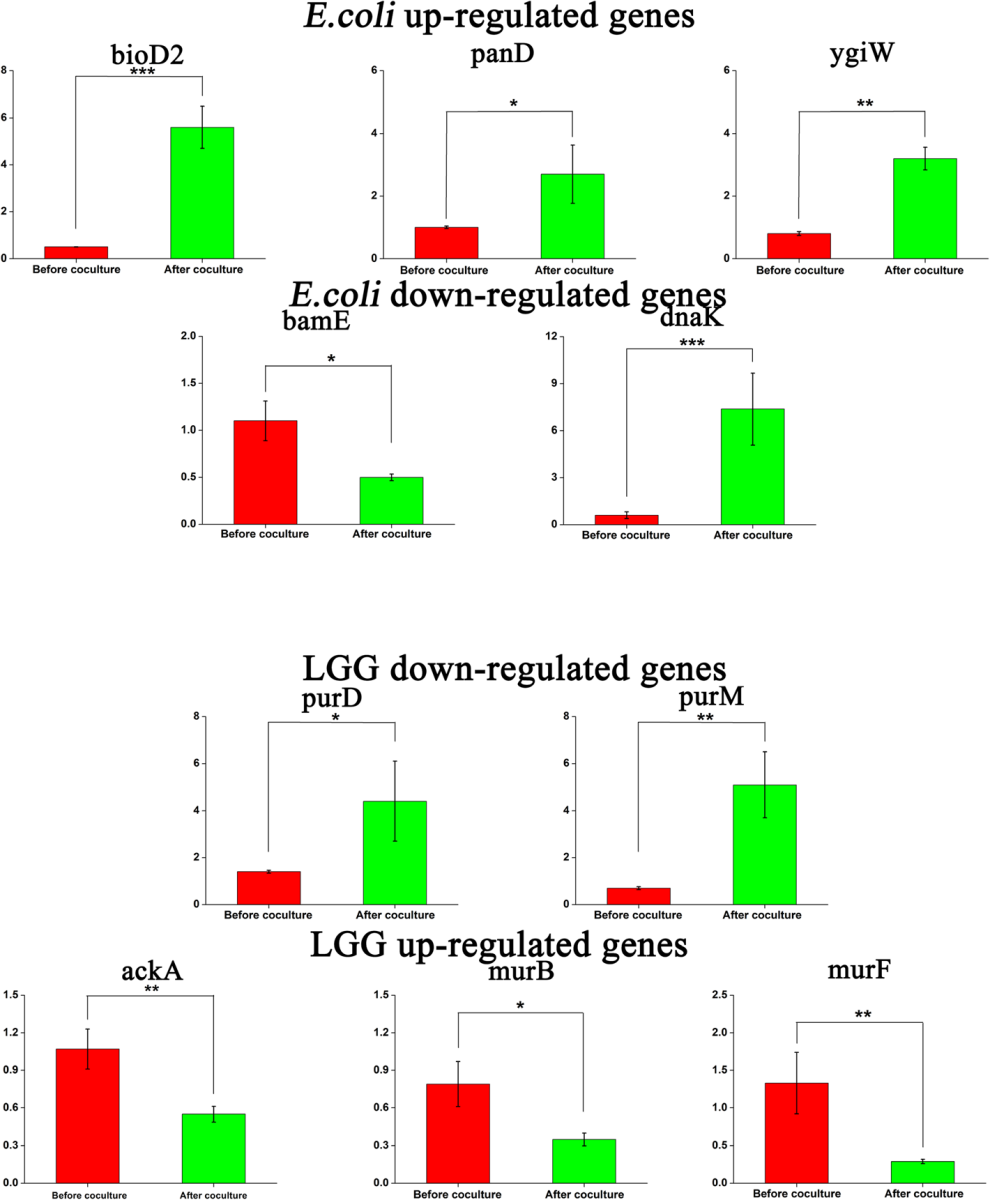
Relative mRNA expression of selected targets from global proteomics analysis. Total RNA isolated from coculture treatment or pure culture of *E. coli* and LGG was reversed transcribed and cDNA was quantified by qRT-PCR using target-specific primers. The data represents the mean ± SD of triplicate experiments normalised to 16S RNA. Statistically significant differences between coculture treatment and pure culture treatment, as determined by Student’s t-test analysis (unpaired, two-tailed), are represented as *p ≤ 0.5, **p ≤ 0.1 and ***p ≤ 0.01

qRT-PCR analysis of selected targets was conducted to validate the observed differentially expressed protein levels (Fig. 5). In line with the findings from the global proteomic analysis, increased *bioD2*, *panD* and *ygiW* mRNA levels in *E. coli* were observed. The mRNA levels of *bamE* and *dnaK*, which encode downregulated expression at the transcription level, were also quantified. Additionally, given the extensive effect of LGG microcapsules on biofilm inhibition, the mRNA expression levels of *purD* and *purM* were found to be upregulated 3.1-fold and 7.3-fold, respectively. The mRNA levels of *murB*, *murF* and *ackA* were also decreased after coculture with *E. coli* for 48 h.

### Antibiotic resistance

**Fig. 6:**
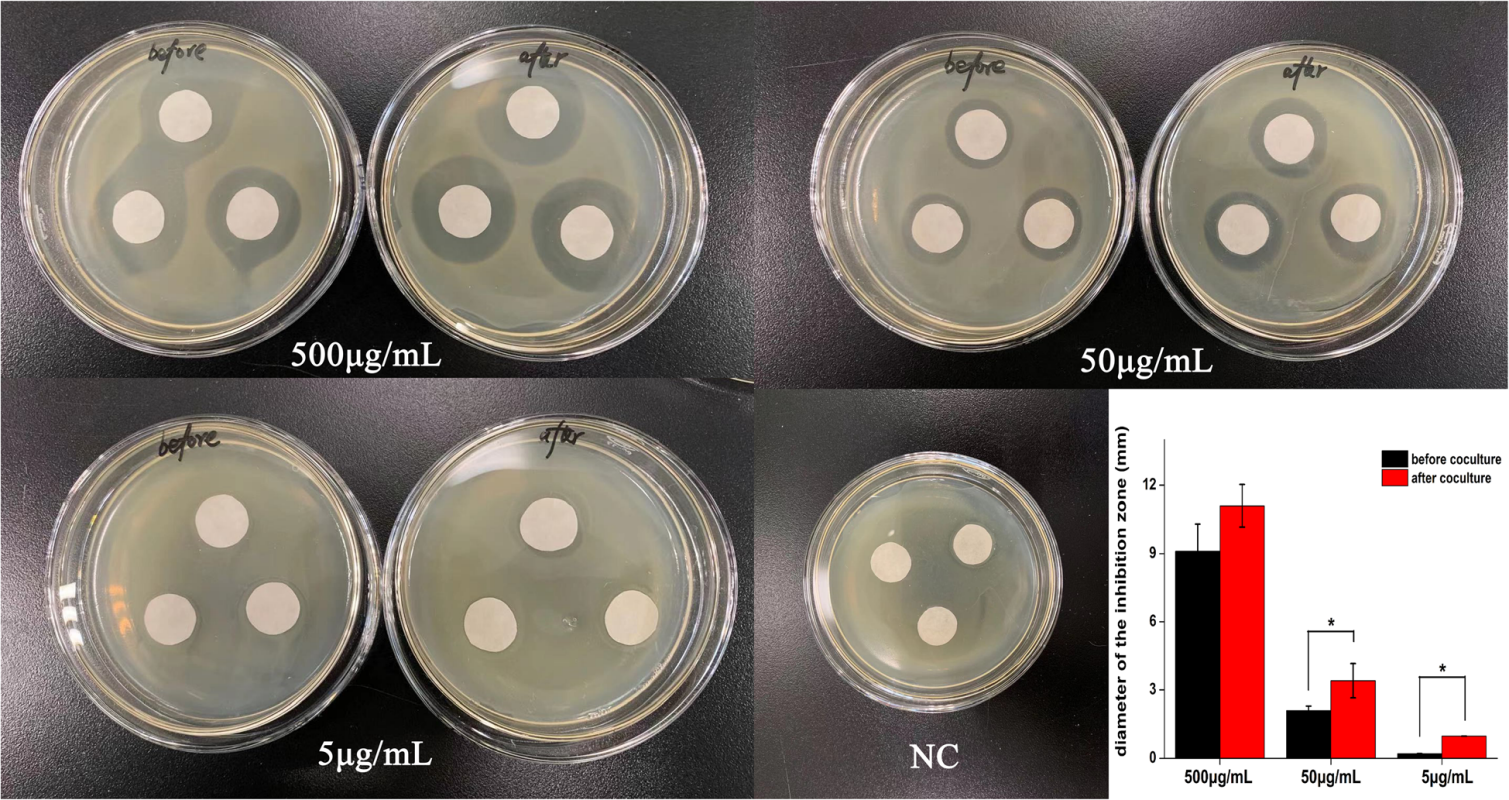
Inhibition zone to evaluate the antibiotic resistance of *E. coli* before and after coculture with LGG microcapsules. Amp was used as the standard antibiotic at 500 μg/mL, 50 μg/mL or 5 μg/mL

The antibiotic resistance of *E. coli* obtained from pure culture and after coculture with LGG microcapsules was determined. The results showed that the *E. coli* antibiotic resistance was concentration-dependent: the inhibition zone of the *E. coli* pure culture was 9.1 ± 1.2 mm, 2.1 ± 0.2 mm and 0.2 ± 0.03 mm at the three decreasing Amp concentrations, respectively; furthermore, the inhibition zone of *E. coli* after coculture was 11.1 ± 0.93 mm, 3.4 ± 0.76 mm and 0.98 ± 0.03 mm, respectively, each of which was significantly larger than the inhibition zone of the *E. coli* pure culture at different Amp concentrations. Negative controls did not show any inhibitory effect on the growth of tested bacteria (Fig. 6). Antibiogram results revealed that *E. coli* after coculture with LGG microcapsules showed increased susceptibility to Amp compared with pure *E. coli* culture.

## Discussion

Biofilm formation is associated with resistance to antibiotic therapy and therefore continues to be a major health threat in both hospital and community settings [15, 16]. We previously reported that probiotic LGG microcapsules could inhibit *E. coli* biofilm formation without causing antibiotic resistance. Probiotics have many benefits for human health and are used both therapeutically and in the food industry [17]. These findings prompted the present evaluation of the mechanism of action underlying the inhibitory effect of LGG microcapsules on antibacterial biofilm formation.

This study demonstrated that, after coculture treatment, there were 1655 and 1431 proteins expressed in *E. coli* and LGG strains, respectively. Overall, 262 and 291 common differentially expressed proteins, respectively, exhibited greater than 2-fold changes in expression compared to the control group.

### Effects of LGG microcapsules on biofilm formation capacity and the metabolism of *E. coli* in coculture

There are four major steps involved in biofilm formation: (i) initial adhesion or attachment (reversible); (ii) early development of biofilm structure (irreversible); (iii) maturation of the developed biofilm; and, (iv) dispersion of cells from the biofilm to return to the planktonic state. Many genes and proteins are involved in the complex process of biofilm formation [18]. Proteomics result also revealed some proteins with functions relating to biofilm formation and development. For example, OmpA protein is associated with biofilm formation [19], and an association between biofilm formation, structure and the expression levels of genes relating to biofilm formation and biofilm-specific resistance was found in *Acinetobacter baumannii* Strains isolated from burn infections in Ahvaz, Iran. Furthermore, some other proteins have been reported in relation to biofilm formation and development, such as Dnak [20] and RpoA [21]. It is assumed that the downregulation of these proteins in *E. coli* after coculture with LGG microcapsules decreased the initial adhesion or attachment ability of *E. coli*, which resulted in decreased biofilm thickness (Fig. 1d).

Proteomic analysis detected differentially expressed *E. coli* proteins before and after coculture. These differences were related to cellular responses to DNA damage stimulus and cell wall organisation. Thus, these findings indicated that the proteins involved in response to the environment changed during coculture. Based on this, the stress response of *E. coli* was focused on in the coculture model. Accordingly, increased mRNA levels of the *bioD2* gene were observed. The bioD2 protein is an ATP-dependent dethiobiotin synthetase that encodes a homolog of dethiobiotin synthetase, which is the penultimate enzyme in the biotin synthesis pathway. Therefore, it is likely that this upregulated *bioD2* expression in the presence of LGG microcapsules enhanced the degrader’s requirement for biotin, which is synthesised de novo under the acidic, osmotic and oxidative stress conditions with the involvement of different isozymes. This explanation was supported by the upregulated expression observed in both the proteomics and qRT-PCR analyses and was further validated by the inhibitory effect of LGG microcapsules on *E. coli* biofilm formation.

Aspartate 1-decarboxylase (PanD) is the only enzyme capable of β-alanine synthesis in *E. coli*. In bacteria, fungi and plants, β-alanine is a precursor to pantothenate which, in turn, is a required metabolite for the synthesis of coenzyme A (CoA) in all organisms [22]. Research indicates that chloroplast engineering of the beta-alanine pathway by overexpression of *E. coli* panD enhances thermotolerance of photosynthesis and biomass production following high-temperature stress [23].

During the coculture process, *E. coli* strains were frequently confronted by acid stress produced by LGG metabolism. The ygiW protein is reported to be involved in the stress response associated with exposure to H_2_O_2_, cadmium and acid [24]. An earlier study also reported that the expression of functional YgiW and QseC proteins is necessary for optimal biofilm growth of *Aggregatibacter actinomycetemcomitans* [25]. Comparison of the expression levels of the *ygiW* gene between LGG microcapsules and coculture conditions revealed a 4.0-fold change.

Proteomic analysis revealed the downregulation of several virulence-related proteins, including *bamE* and *dnaK,* when *E. coli* was treated with LGG microcapsules. The most downregulated protein, bamE (MHC class II analog protein, log2FC = − 9.2), is an integral outer membrane β-barrel protein (OMP) that is assembled by the beta-barrel assembly machine (Bam) complex in Gram-negative bacteria [26]. Another downregulated protein, DnaK, is an important factor in all three antibiotic-related persister formation pathways. The decreased persistence phenotype, as well as the growth defect of *dnaK,* seem to depend on functional (p) ppGpp [27]. Heterogeneous expression of the *dnaK* gene in *Alicyclobacillus acidoterrestris* can significantly enhance the resistance of host bacteria *E. coli* against heat and acid stresses [28]. Furthermore, the DnaK protein has also been reported to play an important role in bacterial biofilm formation [29, 30].

EPS is the characteristic that distinguishes biofilms from planktonic bacteria. The EPS matrix is the medium through which bacterial cells are attached to the surface and facilitate cell-to-cell as well as cell-to-surface interactions. It provides support to biofilm cells and gives the biofilm a three-dimensional architecture, thus providing a protective as well as structural role. Water is one of the major components of the EPS, along with extrapolymeric polymers, proteins, nucleic acids, nutrients, lipids and other metabolites. An EPS inhibition effect has been reported in many papers [31]. However, in the present study no differentially expressed proteins that were downregulated in *E. coli* in the coculture model related to EPS formation.

In summary, it is believed that the LGG microcapsules inhibited *E. coli* biofilm formation and decreased antibiotic resistance mainly through the disruption of cell metabolism and by decreasing the expression of stress-related proteins.

### Effects of *E. coli* on the growth and metabolism of LGG microcapsules in coculture

Proteomic analysis indicated that *E. coli* coculture with LGG microcapsules elicited a cellular response in LGG and *E. coli* strains that was related to a certain intracellular mechanism. Coculture with LGG microcapsules places environmental stress on *E. coli* and this, in turn, raises a cellular response in LGG as well [32]. For LGG, the possible responses to *E. coli* coculture include physiological and developmental changes, reprogramming of the resistance gene or proteins, and alterations to how energy is supplemented. In the present study, proteomic analysis revealed that coculture with *E. coli* significantly upregulated two nucleotide metabolism–related genes, *purD* and *purM*. The *pur*-operon (*purEKCSQLFMNHD*) is responsible for the catalysis of de novo synthesis of inosine monophosphate (IMP) from phosphoribosyl pyrophosphate [33]. In *Staphylococcus aureus,* purine biosynthesis enzymes have been closely implicated in the virulence, persistence and tolerance of stresses such as antibiotic resistance [34, 35]. Such extensive effects could be attributed to the potential modulation of transcription of the operon by bacteria-secreted extracellular compounds. In another study, *purD* and *purF* mutants were constructed in macrophage-like RAW264.7 and HeLa cells. The *purD* and *purF* mutants showed significantly decreased intracellular survival, and complementation of these mutants with intact copies of the *purD* or *purF* genes of *Brucella abortus* strain RB51 restored these defects. These findings suggest that genes encoding the early stages of purine biosynthesis (*purD* and *purF*) are required for intracellular survival and virulence of the RB51 strain [36]. Therefore, it may be that LGG strains maintain intracellular survival and homeostasis by upregulating the *purD* and *purM* genes.

Coculture of LGG microcapsules with *E. coli* appeared to downregulate *murB* gene expression and to completely abolish expression of the *murF* gene. Bacteria generally synthesise their own active form of N-acetylmuramic acid, UDP-N-acetylmuramic acid [37], and the MurB enzyme (UDP-N-acetylglucosamine pyruvate enol ether reductase) plays an important role in the biosynthesis of this substance [38]. The MurB enzyme converts UDP-N-acetylglucosamine pyruvate enol ether to UDP-N-acetylmuramic acid by reducing its double bond [39]. Inhibition of the MurB enzyme reduces or blocks the synthesis of peptidoglycan, resulting in an incomplete bacterial cell wall; this eventually leads to the production of lytic bacteria under the pressure of permeation [40]. Therefore, downregulation of the *murB* and *murF* genes implies suppressed LGG cell membrane biosynthesis, to some extent, when LGG was cocultured with *E. coli*.

Metabolism refers to the basic physiological processes that maintain a living organism. Coculture of LGG microcapsules with *E. coli* was associated with the downregulation of metabolism-related genes. Acetate presumably provides a relevant nutrient for *Enterobacteria* as well as other bacteria [41, 42]. In *E. coli*, the primary pathway of acetate production involves two enzymes that are intimately connected to central metabolism, phosphotransacetylase (Pta) and acetate kinase (AckA) [43]. During exponential growth, acetyl-CoA, the product of glycolysis and the consumable substrate for the tricarboxylic acid (TCA) cycle, can be converted into acetylphosphate (AcP) by Pta and then into acetate by AckA. *E. coli* also takes up acetate, using the Pta-AckA pathway in reverse, resulting in the synthesis of acetyl-CoA. This pathway typically operates at high extracellular acetate concentrations (≥ 8 mM) [44]. Disruption of the Pta-AckA pathway during overflow metabolism causes a significant reduction in the growth rate and viability of the bacteria, although this is not due to intracellular ATP depletion [45, 46]. Hence, downregulation of the *ackA* gene will affect LGG metabolism.

## Conclusions

As far as is known, the present study is the first published attempt to determine protein expression differences associated with a probiotic *E. coli* in situ coculture. Label-free quantitative proteomic analysis indicated that *E. coli* and microencapsulated LGG may impact cellular metabolism, the stress response, transcription, and the cell membrane through regulating the expression of PanD, YgiW, BioD2, DamE and DnaK proteins in *E. coli*, and PurD, PurM, AckA, MurB, MurF and RpoC proteins in LGG. The coculture with LGG microcapsules also decreased the *E. coli* resistance to Amp. Taken together, these findings further understanding of the possible molecular action of LGG microcapsules against *E. coli* biofilm formation. Future studies will focus on the analysis of posttranslational modifications of differentially expressed proteins as well as endogenous protein complexes and protein-protein interactions.

## Supplementary Information

**Additional file 1 **: **Table S1.** Primers used in this study.

**Additional file 2: Figure S1.** Volcano plots of differentially expressed proteins after *E. coli* and LGG microcapsule coculture. Volcano plots were generated based on the fold-change of protein levels using averaged spectral counts from biological triplicates. The x-axis indicates a log2-fold change and the y-axis indicates -log10 *p*-values based on Student’s t-test. The horizontal line indicates a *p*-value < 0.5 and the vertical lines represent a fold-change > 1.5. In all plots, the green dots represent upregulated proteins in the upper left quadrant, whereas downregulated proteins are shown as red dots in the upper right quadrant. Black dots indicate proteins for which differences in abundance were not statistically significant.

**Additional file 3: Table S2.** Most highly differentially upregulated and downregulated proteins in *E. coli* after coculture.

**Additional file 4: Table S3.** Most highly differentially upregulated and downregulated proteins in LGG microcapsules after coculture.

## Data Availability

The datasets generated and/or analysed during the current study are available in the [figshare] repository, [10.6084/m9.figshare.11923542].
