# Supplementary material for: Label-free quantitative proteomic analysis of the inhibition effect of Lactobacillus rhamnosus GG on Escherichia coli biofilm formation in co-culture
Source: Proteome Sci. 2021 Mar 9;19:4. doi: 10.1186/s12953-021-00172-0 (PMC7945214; doi:10.1186/s12953-021-00172-0)
Supplement: Supplementary file 1 — Additional file 1 : Table S1. Primers used in this study. [file 12953_2021_172_MOESM1_ESM.docx]

**Table S1.** Primers used in this study.

| **Stains** | **Genes** | **Oligonucleotide sequence (5＇-3＇)** | **source** |
| --- | --- | --- | --- |
| *E.coli* | *bioD2* | AGGTCTCTAGAGCTCGCGGATTCGATATCCGT | This study |
|  |  | TACTAAGCTTCGCACAAAGCACGATCGGCA |  |

|  | *panD* | AAGGTAGAACATATGATTCGCACGATGCTGCA | This study |
| --- | --- | --- | --- |
|  |  | CCAGCCGCAAGCTTAACAATCAAGCAACCTGT |  |
|  | *ygiW* | CGAATGCGCCGAAAATGGAACACCACAAAAAAGGTG  CAATTTTTTCTACGCGTTCAATATCCACTTCGGTCTTATTCC | This study |
|  | *bamE* | GAATGGCCTGCGCCATGGCCGAGGAAAGGAACG | This study |
|  |  | GATAAAGTACTCGCGCCGGATCCGGCAA |  |
|  | *dnaK* | TTGTAATCTTATGATTTGGTTATTATATCTGTGATTA | This study |
|  |  | AACATTAGAATCTAAACCAATAATATAGACACTAAT |  |
|  | 16sRNA | GGTGGCTAAATGCCGTTGTT | This study |
|  |  | TGCGGGGTGTTCATTGTTT |  |
| LGG | *purD* | GGGGTGCAACATTTTGAATACTGA | This study |
|  |  | TCTTTGCAATGCTTCATCGTT |  |
|  | *purM* | AACTGCACATACACGCTTACA | This study |
|  |  | GGCAATGAGTCTGTGAGATTT |  |
|  | *murB* | CCAATTATTTGGAGAAAGAGGCTAA | This study |
|  |  | GCACAAAAGGATGAAGAGAAAGT |  |
|  | *murF* | ATGTATCGTCAACAACCTATATTTTTGG | This study |
|  |  | ACACAATAAAACAGAATTGAGATAAAGGT |  |
|  | *ackA* | AGC​AGTAGGGAATCTTCC​A | This study |
|  |  | ATTYCACCGCTACACATG |  |

|  | 16sRNA | CCTAYGGGRBGC​ASC​AG | This study |
| --- | --- | --- | --- |
|  |  | GGA​CTA​CNNGGGTAT​CTAAT |  |
