# Supplementary material for: Label-free quantitative proteomic analysis of the inhibition effect of Lactobacillus rhamnosus GG on Escherichia coli biofilm formation in co-culture
Source: Proteome Sci. 2021 Mar 9;19:4. doi: 10.1186/s12953-021-00172-0 (PMC7945214; doi:10.1186/s12953-021-00172-0)
Supplement: Supplementary file 2 — Additional file 2: Figure S1. Volcano plots of differentially expressed proteins after E. coli and LGG microcapsule coculture. Volcano plots were generated based on the fold-change of protein levels using averaged spectral counts from biological triplicates. The x-axis indicates a log2-fold change and the y-axis indicates -log10 p-values based on Student’s t-test. The horizontal line indicates a p-value < 0.5 and the vertical lines represent a fold-change > 1.5. In all plots, the green dots represent upregulated proteins in the upper left quadrant, whereas downregulated proteins are shown as red dots in the upper right quadrant. Black dots indicate proteins for which differences in abundance were not statistically significant. [file 12953_2021_172_MOESM2_ESM.docx]

**
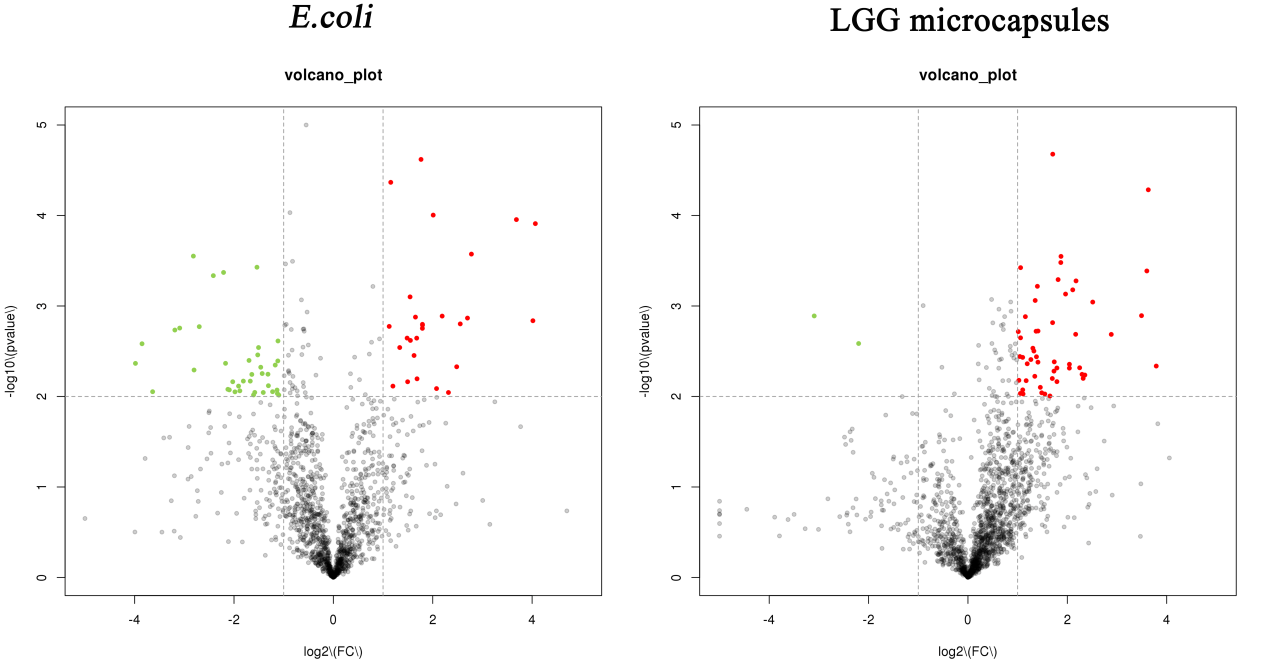
**

**Figure S1.** Volcano plots of differentially expressed proteins after *E.coli* and LGG microcapsules coculture. Volcano plots were generated based on fold-change of protein levels using the averaged spectral counts from biological triplicates. The x-axis indicates a log2 fold-change, and the y-axis indicates -log10 *p*-value based on Student’s t test. The horizontal line indicates a *p*-value < 0.05, and the vertical lines represent a fold-change > 1.2. In all plots, the green dots represent the up-regulated proteins in the upper left quadrant, whereas those that were downregulated proteins are shown as red dots in the upper right quadrant. Black dots indicate proteins for which differences in abundance were not statistically significant.
