# Supplementary material for: Label-free quantitative proteomic analysis of the inhibition effect of Lactobacillus rhamnosus GG on Escherichia coli biofilm formation in co-culture
Source: Proteome Sci. 2021 Mar 9;19:4. doi: 10.1186/s12953-021-00172-0 (PMC7945214; doi:10.1186/s12953-021-00172-0)
Supplement: Supplementary file 3 — Additional file 3: Table S2. Most highly differentially upregulated and downregulated proteins in E. coli after coculture. [file 12953_2021_172_MOESM3_ESM.docx]

**Table S2.** Most highly common differentially upregulated and downregulated proteins in *E.coli*

| **Upregulated protein** | **Protein ID** | ***p* value** | **Log2FC** | **Protein description** |
| --- | --- | --- | --- | --- |
| **bioD2** | P0A6E9 | 2.10E-05 | 1.71 | ATP-dependent dethiobiotin synthetase BioD 2 |
| **panD** | P0A790 | 5.20E-05 | 3.63 | Aspartate 1-decarboxylase |
| **rpsP** | P0A7T3 | 2.83E-04 | 1.86 | 30S ribosomal protein S16 |
| **hybC** | P0ACE0 | 3.31E-04 | 2.01 | Hydrogenase-2 large chain |
| **rhlB** | P0A8J8 | 3.77E-04 | 1.06 | ATP-dependent RNA helicase RhlB |
| **mlaC** | P0ADV7 | 4.10E-04 | 3.60 | Intermembrane phospholipid transport system binding protein MlaC |
| **fimA** | P04128 | 5.11E-04 | 1.81 | Type-1 fimbrial protein, A chain |
| **hdeA** | P0AES9 | 7.83E-04 | 1.17 | Acid stress chaperone HdeA |
| **cysQ** | P22255 | 1.89E-03 | 1.41 | 3'(2'),5'-bisphosphate nucleotidase CysQ |
| **ygiW** | P0ADU5 | 2.06E-03 | 2.17 | Protein YgiW |
| **Downregulated protein** | **Protein ID** | ***p* value** | **Log2FC** | **Protein description** |
| **bamE** | P0A937 | 9.90E-04 | -0.90 | Outer membrane protein assembly factor BamE |
| **yejL** | P0AD24 | 1.29E-03 | -3.09 | UPF0352 protein YejL |
| **rpsN** | P0AG59 | 2.60E-03 | -2.17 | 30S ribosomal protein S14 |
| **gpmA** | P62707 | 4.76E-03 | -0.52 | 2,3-bisphosphoglycerate-dependent phosphoglycerate mutase |
| **dnaK** | P0A6Y8 | 9.35E-03 | -0.27 | Chaperone protein DnaK |
| **sohB** | P0AG14 | 1.43E-02 | -0.45 | Probable protease SohB |
| **ychF** | P0ABU2 | 1.63E-02 | -0.35 | Ribosome-binding ATPase YchF |
| **guaB** | P0ADG7 | 2.64E-02 | -0.97 | Inosine-5'-monophosphate dehydrogenase |
| **ftsX** | P0AC30 | 3.38E-02 | -2.46 | Cell division protein FtsX |
| **ompA** | P0A910 | 4.93E-02 | -1.08 | Outer membrane protein A |
