# Supplementary material for: Label-free quantitative proteomic analysis of the inhibition effect of Lactobacillus rhamnosus GG on Escherichia coli biofilm formation in co-culture
Source: Proteome Sci. 2021 Mar 9;19:4. doi: 10.1186/s12953-021-00172-0 (PMC7945214; doi:10.1186/s12953-021-00172-0)
Supplement: Supplementary file 4 — Additional file 4: Table S3. Most highly differentially upregulated and downregulated proteins in LGG microcapsules after coculture. [file 12953_2021_172_MOESM4_ESM.docx]

**Table S3.** Most highly common differentially upregulated and downregulated proteins in LGG

| **Upregulated protein** | **Protein ID** | ***p* value** | **Log2FC** | **Protein description** |
| --- | --- | --- | --- | --- |
| **CCE29_04955** | A0A1Y0DVK9 | 2.40E-05 | 1.76 | Pilus assembly protein |
| **LRHMDP2_922** | K8QM21 | 4.30E-05 | 1.15 | NADPH:quinone reductase related Zn-dependent oxidoreductase |
| **CCE29_07950** | A0A1Y0DXE7 | 9.90E-05 | 2.01 | Iron-sulfur cluster biosynthesis family protein |
| **AAULR_10650** | F3N0G5 | 1.11E-04 | 3.68 | Membrane protein |
| **CCE29_03735** | A0A2A5L8F8 | 1.23E-04 | 4.07 | ABC transporter substrate-binding protein |
| **CCE29_04965** | A0A1Y0DVP1 | 2.67E-04 | 2.78 | Pilus assembly protein |
| **N507_1524** | A0A249N5Y8 | 6.08E-04 | 0.80 | Uncharacterized protein |
| **LRHMDP2_518** | K8QF03 | 7.93E-04 | 1.54 | Uncharacterized protein |
| **purD** | A0A1Y0DZS2 | 1.60E-03 | 1.79 | Phosphoribosylamine--glycine ligase |
| **purM** | A0A1Y0DZP0 | 1.63E-03 | 1.79 | Phosphoribosylformylglycinamidine cyclo-ligase |
| **Downregulated protein** | **Protein ID** | ***p* value** | **Log2FC** | **Protein description** |
| **rpoC** | K8QEG2 | 8.00E-06 | -0.55 | DNA-directed RNA polymerase subunit beta' |
| **secA** | A0A249DE52 | 9.30E-05 | -0.87 | Protein translocase subunit SecA |
| **murB** | A0A3S4R547 | 2.81E-04 | -2.82 | UDP-N-acetylenolpyruvoylglucosamine reductase |
| **murF** | A0A2A5L3G4 | 3.21E-04 | -0.82 | UDP-N-acetylmuramoyl-tripeptide--D-alanyl-D-alanine ligase |
| **rplV** | K8Q8J1 | 3.43E-04 | -0.96 | 50S ribosomal protein L22 |
| **ddl** | A0A1Y0DWH7 | 3.73E-04 | -1.54 | D-alanine--D-alanine ligase |
| **LRHMDP2_1796** | K8Q7G6 | 4.26E-04 | -2.21 | 6-phospho-beta-glucosidase |
| **N507_1229** | A0A249N444 | 4.62E-04 | -2.42 | 50S ribosomal protein L16 |
| **ackA** | K8Q7E5 | 8.56E-04 | -0.64 | Acetate kinase |
| **lpdA** | A0A1Y0DTI9 | 1.75E-03 | -1.08 | Outer membrane protein A |
